# Predictive factors of Osaka Medical College (OMC) brace treatment in patients with adolescent idiopathic scoliosis

**DOI:** 10.1186/s13013-015-0038-7

**Published:** 2015-04-10

**Authors:** Hiroshi Kuroki, Naoki Inomata, Hideaki Hamanaka, Kiyoshi Higa, Etsuo Chosa, Naoya Tajima

**Affiliations:** Department of Orthopaedic Surgery, National Hospital Organization Miyazaki Higashi Hospital, 4374-1 Tayoshi Ooaza, Miyazaki, 880-0911 Japan; Department of Orthopaedic Surgery, University of Miyazaki Faculty of Medicine, Miyazaki, Japan; Department of Orthopaedic Surgery, Nozaki Higashi Hospital, Miyazaki, Japan

**Keywords:** Adolescent idiopathic scoliosis (AIS), Osaka Medical College (OMC) brace, Conservative treatment, Predictive factor, Standardized inclusion and assessment criteria, Scoliosis Research Society (SRS)

## Abstract

**Background:**

Factors influencing clinical course of brace treatment apply to adolescent idiopathic scoliosis (AIS) patients remain unclear. By making clear them, we may select suitable patients for brace treatment and alleviate overtreatment. The purpose of this study was to explore predictive factors of Osaka Medical College (OMC) brace treatment for AIS patients in accordance with the modified standardized criteria proposed by the Scoliosis Research Society (SRS) committee on bracing and non-operative management.

**Methods:**

From 1999 through 2010, 31 consecutive patients with AIS who were newly prescribed the OMC brace and met the modified SRS criteria were studied. The study included 2 boys and 29 girls with a mean age of 12 years and 0 month. We investigated the clinical course and evaluated the impacts of compliance, initial brace correction rate, curve flexibility, curve pattern, Cobb angle, chronological age, and Risser stage to clinical outcomes. The clinical course of the brace treatment was considered progression if ≥6° curvature increase occurred and improvement if ≥6° curvature decrease occurred according to SRS judgment criteria.

**Results:**

The curve progressed in 10 cases, the curve improved in 6 cases, and the curve remained unchanged in 15 cases (success rate: 67.7%). The success rate was statistically higher in the patient group whose instruction adherence rate was greater than 50% as compared with in those 50% or less. Initial brace correction rate, curve flexibility, curve pattern, the magnitude of Cobb angle, chronological age, and Risser stage did not have any significant effect for clinical courses. However, success rate was insignificantly higher in the cases whose Cobb angle in brace was smaller than that in hanging position.

**Conclusions:**

OMC brace treatment could alter the natural history of AIS, however, that was significantly affected by compliance of brace wear.

## Introduction

Current treatment for adolescent idiopathic scoliosis (AIS) is divided into operative treatment and non-operative treatment. In the past, various non-operative treatments, including exercise, physical therapy, electrical stimulation, and brace treatment have been tried to delay or prevent the curve progression. Of those, brace treatment is the only widely accepted and demonstrated the efficacy to alter the natural history of AIS [1,2]. Recently, Lange et al. [3] reported that long-term results at a mean of 19.2 years were also satisfactory in most patients with AIS treated with the Boston brace. However, some physicians have raised serious questions about the efficacy of brace to stop deterioration of the deformity [4-6].

**Figure 1:**
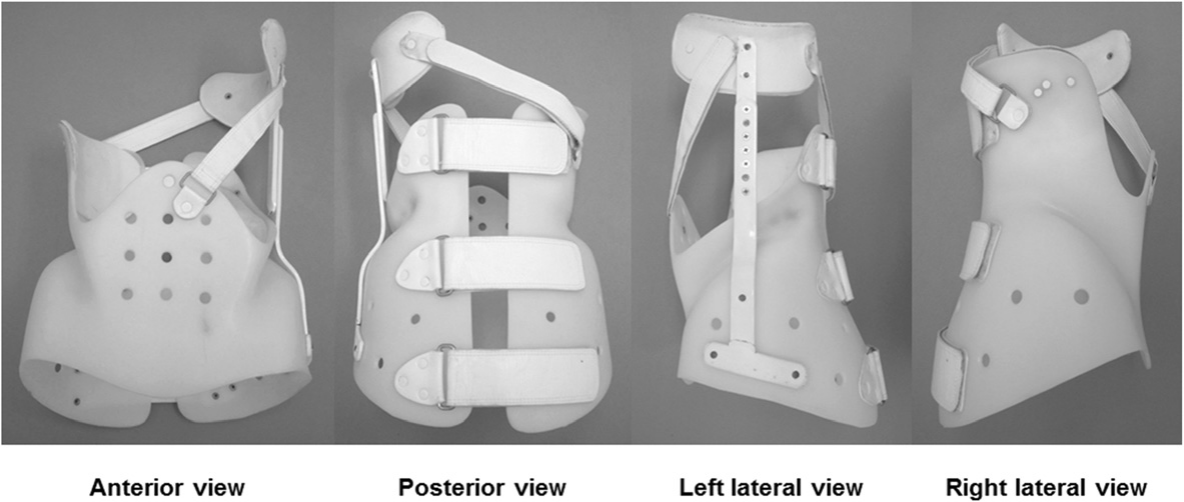
**Osaka Medical College (OMC) brace.** The OMC brace simply consists of a pelvic girdle, an upright bar, a high thoracic pad, and two straps. A pelvic girdle facilitates correction of a main thoracic curve. An upright bar with a high thoracic pad can correct a proximal thoracic curve utilizing righting reflex produced by patients’ own response. Anterior and posterior straps prevent the body moving away from the brace during forward and backward bending motion.

One of the large issues to disturb the resolution of this argument is wide differences in the documented outcomes of various studies. Heterogeneity of the study samples has been proposed as a reason for the conflicting results [7]. Then, the Scoliosis Research Society (SRS) has standardized criteria for brace studies in patients with AIS to make comparisons among studies more valid and reliable [8]. We carried out a clinical research to evaluate the effectiveness of Osaka Medical College (OMC) brace treatment using the modified SRS standardized criteria, and obtained the results that it could halt scoliosis progression and reduced the need for surgery [9]. The OMC brace is one of the popular custom-made thoracolumbosacral orthoses (TLSOs) in Japan developed by Onomura in 1970’s [10]. The characteristics of the OMC brace are represented by inconspicuous design, light weight, reduction of restriction on the chest wall movement, and ability to correct the high thoracic curve by righting reflex [10]. The concept of this brace is maintenance of whole body alignment and balance. For the achievement of these goals, step-by-step molding from pelvic girdle to high thoracic level with correcting lumbar and main thoracic curves is important to generate desirable corrective force based on the principle of three points lateral compression [11] (Figure 1).

Another major issue of brace treatment for AIS is about the selection of appropriate patients. It is important that we try to delineate more clearly the indications for brace treatment and avoid overtreatment. Although brace wear in AIS patients does not have any serious physiological side effects, it carries financial, emotional, and social loads that need to be considered [12]. Vijvermans et al. [13] also raise an alarm that by prescribing a brace for an adolescent girl or boy, physicians place literally and metaphorically a burden on their back, in a very important period of their lives. However, factors influencing clinical course of brace treatment apply to AIS patients remain unclear. By making clear which are factors determining the outcome, hoping that taking these factors into account might help physicians to narrow the field of indications of brace treatment [13].

The aim of this study was to explore predictive factors of OMC brace treatment for AIS patients in accordance with the new standardized criteria proposed by the SRS committee on bracing and non-operative management.

## Materials and methods

### Study overview

Medical records and plain x-rays of patients with AIS who were treated with the OMC brace were retrospectively reviewed. To be included in this study, patients met the following criteria: diagnosis of AIS with radiological confirmation of absence of significant pathological malformation of the spine, age 10 years and older when their brace was prescribed, Risser 0–2, primary curve magnitude from 20 to 40°, no prior treatment, and if female, either premenarchal or <1 year postmenarchal. Although primary curve magnitude from 25 to 40° was expressed in SRS criteria, it was our treatment protocol to include immature patients who have progressive scoliosis from 20 to 40°, as a result we employed patients with Cobb angles from 20 to 24°, which is in accordance with a principle of Weinstein et al. [14]. Further, in SRS criteria, only patients who completed their brace treatment and were followed for more than 2 years after skeletal maturity were included. However, Negrini et al. [15] reported the fact that only 85% of patients reached the 2 years follow-up, but this subgroup was not different from the entire population for any basal characteristic nor any final result in their study. For this reason, we employed a follow-up of within 2 years beyond skeletal maturity to increase the number of patients available.

### Patient population

From 1999 through 2010, 31 consecutive patients with AIS who were newly prescribed the OMC brace were studied. The study included 2 boys and 29 girls ranging in age from 10 years and 10 months to 14 years and 8 months, with a mean age of 12 years and 0 month. The type of curves consisted of thoracic in 4 cases, thoracolumbar in 4 cases, lumbar in 12 cases, double major in 7 cases, double thoracic in 1 case, and triple major in 3 cases. Risser stage were grade 0 in 20 cases, grade 1 in 5 cases, and grade 2 in 6 cases. Apexes of main curves were all lower than T7 (T8 in 5 cases, T9 in 6 cases, T10 in 3 cases, T11 in 1 case, T12 in 3 cases, L1 in 1 case, L2 in 8 cases, and L3 in 4 cases). The mean pre-brace Cobb angle of main curves was 27.3°, with the range from 21° to 36°. The duration of brace treatment was from 3 years and 1 month to 6 years and 10 months, with a mean period of 4 years and 8 months. Of these, during brace wear follow-up times were from 1 year and 10 month to 6 years and 1 month, with a mean period of 3 years and 4 months. And post brace weaning follow-up times were from 0 month to 3 years and 3 months, with a mean period of 1 year and 4 months.

### Data collection and analysis

We investigated the clinical course and evaluated the impacts of compliance, initial correction rate by brace wear, curve flexibility, curve pattern, Cobb angle before treatment, chronological age, and Risser stage to clinical outcomes. The estimated number of hours of brace wear in each patient was monitored by self-statement. And the instruction adherence rate was calculated to express the compliance. Curve flexibility was recognized by the flexibility index utilized hanging total spine x-ray [11]. The initial correction rate, the flexibility index, and the instruction adherence rate were conducted by formulae as described below.

- Initial correction rate (%) = {(Cobb angle in upright position - Cobb angle on initial brace wear)/Cobb angle in upright position} × 100
- Flexibility index (%) = {(Cobb angle in upright position - Cobb angle in hanging position)/Cobb angle in upright position} × 100
- Instruction adherence rate (%) = (number of times to visit the outpatient clinic when the patient declared that he or she could wear the brace more than instructed hours minus 2 hours/all number of times to visit the outpatient clinic) × 100

The clinical outcome was assessed based on the SRS criteria. According to the Cobb angle on standing anteroposterior spine x-rays that made with the patients out of the brace were classified as: (1) improved: decrease of the Cobb angle by 6° or more, (2) stable: no more than 5° of progression or improvement, and (3) progressed: increase of the Cobb angle by 6° or more, and (4) progression beyond the Cobb angle of 45° who were considered candidates for surgery. All radiographic measurements were made by 1 author using the same protractor to minimize inter-observer variability in accordance with a concept of Lee et al. [16].

Statistical analyses were defined using a two-tailed paired t-test and chi square test. A value of P < 0.05 was considered statistically significant.

### Brace management protocol

All OMC braces were fabricated by the same certified orthotist. A plaster cast was taken to capture the body shape of each patient, which was used by the certified orthotist to custom make each OMC brace. Then, not only the optimal correction but also comfortable fit and function consisted of pressure pad placements were ensured. Also, standing anteroposterior and lateral spine x-rays were used to check the in brace correction and whole spinal alignment including pelvis while the brace was being worn.

Patients were instructed to wear the brace for a minimum of 20 hours per day at the beginning of brace treatment. When skeletal maturity was noted, that is, all of the following three criteria were fulfilled; a Risser stage of 4, at least 2 years passed since the onset of menstruation (for girls), two consecutive visits over a time period of at least 1 year with no more than a 1-cm increase in height, the brace weaning started and the time in brace was slowly reduced during 1 year. Finally the brace wearing was stopped at 1 year post skeletal maturity.

### Consent

The study was approved by the University’s Institutional Review Board and written informed consent was obtained from the patients and their parents prior to participating. And all procedures were in accordance with the Helsinki declaration.

## Results

The average Cobb angle in upright position before treatment was 27.3 ± 4.2° and that at final follow-up was 28.6 ± 11.3°. As a result, the OMC brace could prevent the progression of curves during periods of growth. At final follow-up, the curve progressed in 10 cases, the curve improved in 6 cases, and the curve remained unchanged in 15 cases. The success rate of 67.7% (21/31) was achieved although the average instruction adherence rate was only 53.7%.

**Figure 2:**
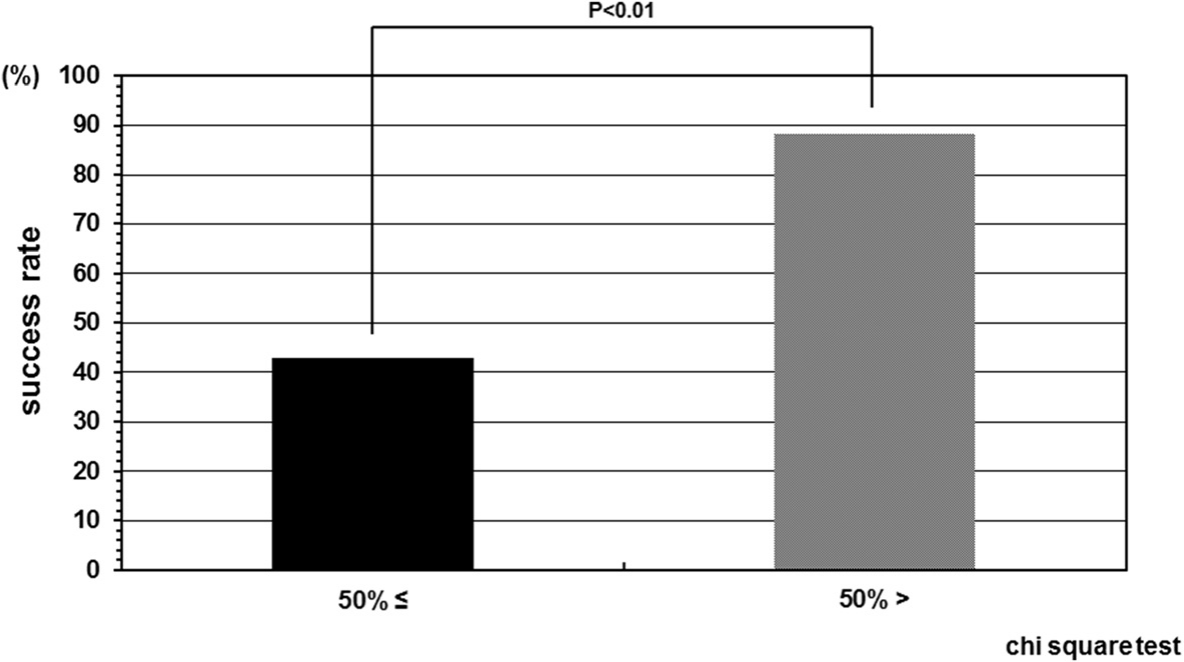
**Success rate depend on compliance.** The success rate in the patient group whose instruction adherence rate was greater than 50% and 50% or less were 88.2% and 42.8%, respectively. The success rate was statistically higher in the patient group whose instruction adherence rate was greater than 50% as compared with in those 50% or less.

**Figure 3:**
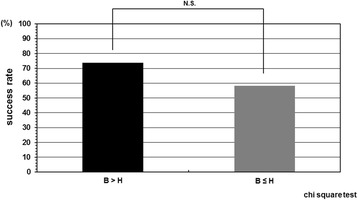
**Success rate depend on relation between Cobb angle in brace wear and that in hanging position.** Success rate was insignificantly higher in the cases whose Cobb angle in brace wear was smaller than that in hanging position although initial correction rate and curve flexibility did not independently affect the clinical results of brace treatment. B: Cobb angle in brace wear, H: Cobb angle in hanging position.

**Figure 4:**
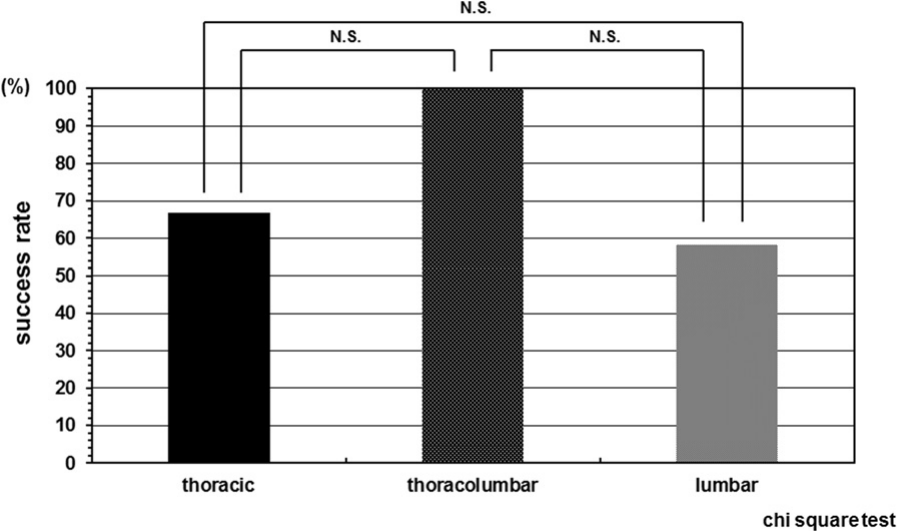
**Success rate depend on curve pattern.** Curve pattern did not have any significant effect for clinical courses.

**Figure 5:**
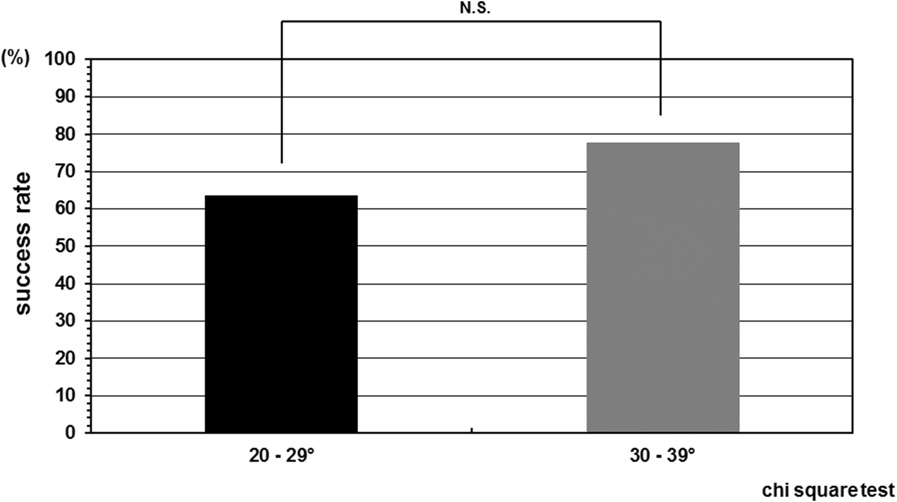
**Success rate depend on Cobb angle before treatment.** Success rate of 20s° was insignificantly lower than 30s°.

**Figure 6:**
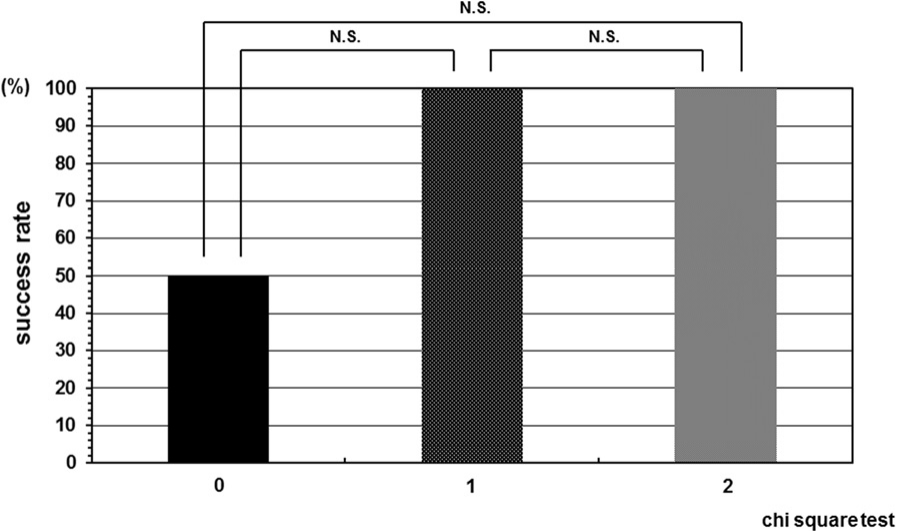
**Success rate depend on Risser stage.** Risser stage did not have any influence for clinical results of brace treatment.

The cases were divided in two groups according to the instruction adherence rate, and as a result, the success rate was statistically higher in the patient group whose instruction adherence rate was greater than 50% compared with in those 50% or less. The success rate in the patient group whose instruction adherence rate was 50% or less was 42.8% (6/14). In contrast, the success rate in the patient group whose instruction adherence rate was greater than 50% was 88.2% (15/17) (Figure 2). Initial correction rate and curve flexibility did not affect the clinical results of brace treatment. However, success rate was insignificantly higher in the cases whose Cobb angle in brace was smaller than that in hanging position (Figure 3). Although curve pattern did not have any significant effect for clinical courses either, success rate of thoracolumbar curves tended to be higher than those of thoracic and lumbar curves (Figure 4). With respect to the magnitude of Cobb angle before treatment, success rate of 20s° was insignificantly lower than 30s° (Figure 5). Both chronological age and Risser stage did not have any influence for clinical results of brace treatment (Figure 6). However, Risser stage of all 10 cases in progression group was grade 0.

## Discussion

Brace treatment is characterized as one of the mainstays of AIS management. However, it is an undeniable fact that the effect of brace varies markedly depending on the patients. In the Bracing in AIS Trial (BRAIST) reported by Weinstein et al. [17], 48% of the untreated patients had a successful outcome. From this viewpoint, Carragee et al. [12] pointed out that the indications of brace treatment described are probably broad, resulting in what may be unnecessary treatment for many patients. In our series, 42.8% in the patient group whose instruction adherence rate was 50% or less did not have cure progression, too. To prescribe a brace to invalid AIS patients means a futile intervention. Therefore, we must make a great effort to select suitable patients for brace treatment and alleviate overtreatment.

Various predictive factors of brace treatment for AIS, such as compliance, curve magnitude before brace prescription, curve pattern, maturity, chronological age, body habitus have been indicated by several reports, so far. Sun et al. [18] found that patients with a younger age, low Risser stage, pre-menarche status, large curve magnitude more than 30°, and main thoracic curve were at high risk of curve progression and requirement for surgery, despite brace treatment in AIS girls. Such a finding was consistent with others previously reported. Vijvermans et al. [13] stated that good results were achieved in older children, with low Cobb angles, and advanced maturity. Lonstein et al. [19] reported that larger curve magnitude before brace initiation was found to be a risk factor for curve progression. With respect to influence on brace outcome of curve pattern, both Soucacos et al. [20] and Ylikoski et al. [21] stated that a right thoracic curve at the highest risk of progression. O’Neill et al. [22] suggested that overweight patients with AIS will have greater curve progression and less successful results following brace treatment than those who are not overweight. Rahman et al. [23] firstly reported the correlation between objective compliance and efficacy. Katz et al. [24] also investigated brace wear control of curve progression in AIS managed with a Boston brace with a heat sensor. And they proved that the greater number of brace wear correlated with not only lack of curve progression but also avoidance for surgical treatment. More recently, Weinstein et al. mentioned the benefit of bracing increased with longer hours of brace wear. Karol [25] stated that bracing is less successful in boys than girls because compliance is problematic in boys.

In current study, only compliance had deep relevance to prognosis of brace treatment for AIS. The success rate was statistically higher in the patient group whose instruction adherence rate was greater than 50% as compared with in those 50% or less. We previously proved that compliance has a tendency to diminish with time, especially in the period when changing in environment like a time proceeding to the next stage of education as a turning point [26]. Therefore, encouragement to patients at this period is important to maintain brace wear as scheduled.

Besides compliance, degree of initial curve correction by brace wear may be a meaningful factor. In current study, success rate was insignificantly higher in the cases whose Cobb angle in brace was smaller than that in hanging position. This result might be related to the low number of cases. If more patients can be enrolled and investigated, significant difference may be revealed. Hence, we consistently aim to prescribe an OMC brace which can correct the curve more in brace than in hanging position. Sponseller [27] set the goal that is 30% in brace correction of thoracic curves and 50% in brace correction of lumbar curves when prescribing any type of brace.

Furthermore, the fact that 10 cases in progression were all grade 0 in Risser stage should not be ignored. This means that more cautious attention has to be paid for more immature AIS patients during brace treatment.

In 2010, Ward et al. [28] introduced DNA-based prognostic testing to predict spinal curve progression in AIS. They concluded that prognostic testing may reduce unnecessary treatments and may lower direct and indirect costs-of-care in low-risk patients with mild AIS. In fact in 2014, Bohl et al. [29] reported a genetic test could predict success for AIS on Providence brace treatment. Selecting suitable AIS patients for brace treatment on the basis of genetic test may be positively considered to avoid overtreatment.

This study has some limitations. First, this is a retrospective observational study without controls. Second, all curves were grouped together. Third, there were a relatively small number of patients. Fourth, long- term follow up was missing. Fifth, compliance of the patients was low, that is, the average instruction adherence rate of our subject was only 53.7% although it was subjectively obtained due to the lack of an objective compliance monitor in the brace. Sixth, the current research was not completely derived from the original SRS criteria. They might influence the results of this research. If a prospective long-term study with larger sample size consisted of sufficient compliant patients will be done, more prognostic factors to be applied suitable patient selection for brace treatment may be conducted. However, we believe that our study will contribute some improvement for the future management of AIS because accumulation of these minor data based on clinical experiences from a great number of institutions must be essential to the future solution of issues around brace treatment for AIS.

In conclusion, OMC brace treatment could alter the natural history of AIS, however, that was significantly affected by compliance of brace wear. To accomplish the acceptable result of brace treatment for AIS, we have to not only prescribe proper braces but also instruct patients about the importance of brace wear to keep their motivation for treatment.
